# Learned end-to-end high-resolution lensless fiber imaging towards real-time cancer diagnosis

**DOI:** 10.1038/s41598-022-23490-5

**Published:** 2022-11-07

**Authors:** Jiachen Wu, Tijue Wang, Ortrud Uckermann, Roberta Galli, Gabriele Schackert, Liangcai Cao, Juergen Czarske, Robert Kuschmierz

**Affiliations:** 1 Laboratory of Measurement and Sensor System Technique, TU Dresden, 01069 Dresden, Germany; 2 State Key Laboratory of Precision Measurement Technology and Instruments, Department of Precision Instruments, Tsinghua University, Beijing, 100084 China; 3 Department of Neurosurgery, University Hospital Carl Gustav Carus, TU Dresden, Dresden, Germany; 4 Division of Medical Biology, Department of Psychiatry, Faculty of Medicine, University Hospital Carl Gustav Carus, TU Dresden, Dresden, Germany; 5 Department of Medical Physics and Biomedical Engineering, Faculty of Medicine Carl Gustav Carus, TU Dresden, Dresden, Germany; 6 Competence Center BIOLAS, TU Dresden, Dresden, Germany; 7 Excellence Cluster Physics of Life, TU Dresden, Dresden, Germany; 8 Faculty of Physics, School of Science, TU Dresden, Dresden, Germany; 9 Else Kröner Fresenius Center for Digital Health, TU Dresden, Dresden, Germany

**Keywords:** Imaging and sensing, Microendoscopy, Cancer imaging

## Abstract

Recent advances in label-free histology promise a new era for real-time diagnosis in neurosurgery. Deep learning using autofluorescence is promising for tumor classification without histochemical staining process. The high image resolution and minimally invasive diagnostics with negligible tissue damage is of great importance. The state of the art is raster scanning endoscopes, but the distal lens optics limits the size. Lensless fiber bundle endoscopy offers both small diameters of a few 100 microns and the suitability as single-use probes, which is beneficial in sterilization. The problem is the inherent honeycomb artifacts of coherent fiber bundles (CFB). For the first time, we demonstrate an end-to-end lensless fiber imaging with exploiting the near-field. The framework includes resolution enhancement and classification networks that use single-shot CFB images to provide both high-resolution imaging and tumor diagnosis. The well-trained resolution enhancement network not only recovers high-resolution features beyond the physical limitations of CFB, but also helps improving tumor recognition rate. Especially for glioblastoma, the resolution enhancement network helps increasing the classification accuracy from 90.8 to 95.6%. The novel technique enables histological real-time imaging with lensless fiber endoscopy and is promising for a quick and minimally invasive intraoperative treatment and cancer diagnosis in neurosurgery.

## Introduction

**Figure 1:**
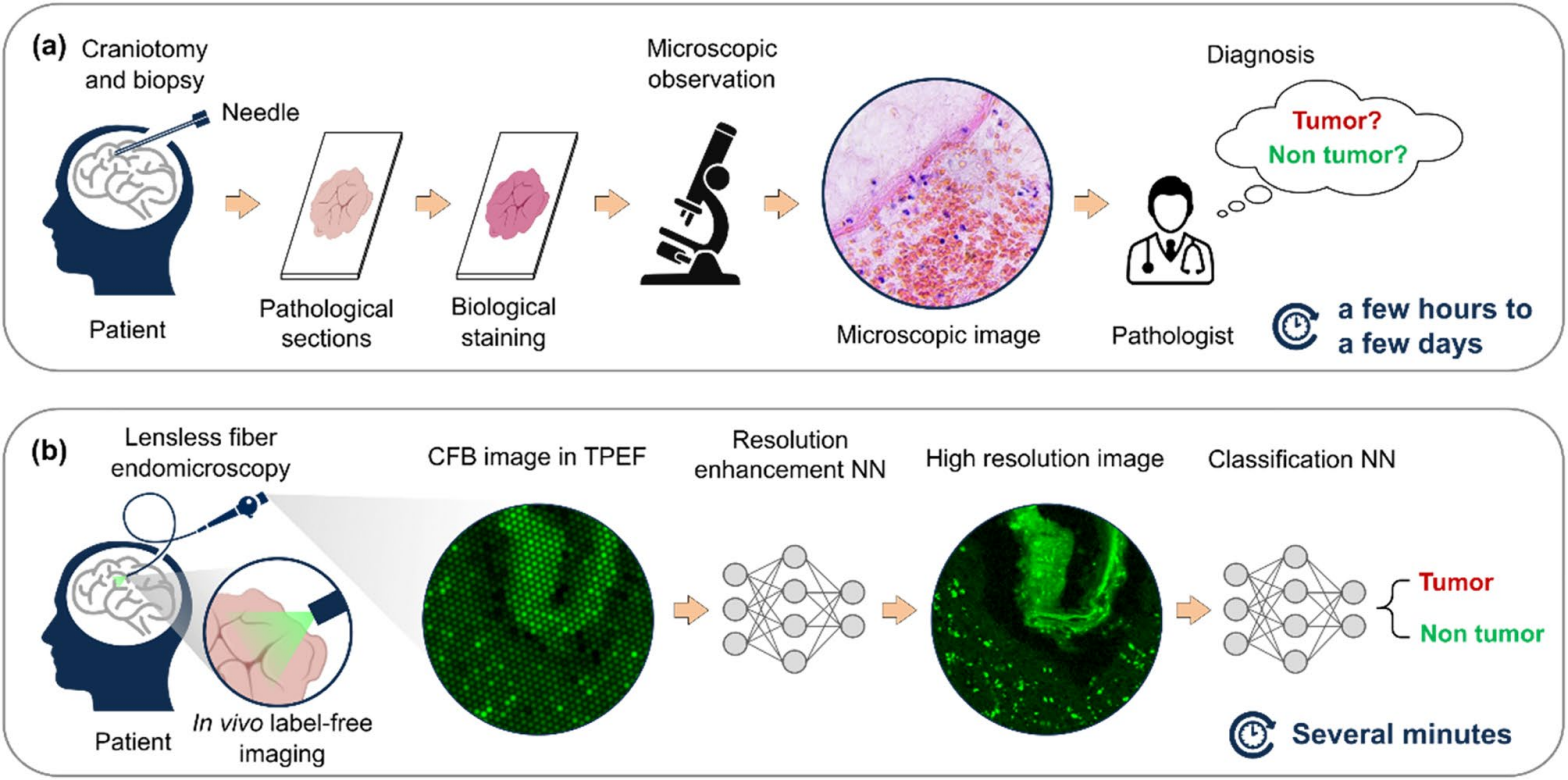
Workflow of biopsy diagnosis and end-to-end diagnosis. (**a**) A biopsy is the main way doctors diagnose most types of cancer diagnosis. It requires specialized surgical skills, histology laboratory and trained personnel. The whole procedure is cumbersome and time-consuming. (**b**) The learning-based end-to-end diagnosis directly obtains the tissue image via minimally invasive fiber-optic endoscope. Deep neural network (DNN) improves resolution for reliable prediction of whether the tissue is a tumor. It is promising for real-time and in situ tumor diagnosis.

Early diagnosis of cancer is the key to improve the survival rate and cure rate of patients. Endoscopy plays an important role in the early stage of cancer diagnosis because there is great benefit in guiding biopsy extraction by histopathological examination. The procedure of biopsy requires sectioning tissues from organs, staining and observation, where pathologists exercise judgment with microscopic images of stained tissue based on their knowledge and experience (Fig. 1a). However, this takes conventionally several hours to a few days, which means, two surgeries are required, the first one for biopsy and the second for tumor resection. This prevents improvement in survival rates, especially for highly aggressive tumors. Moreover, multiple surgical resections lead to increased risk of internal bleeding. To reduce this risk and shorten time for diagnosis, a new approach providing real-time diagnosis is urgently needed. As a key element for implementing multimodal imaging under in vivo conditions, the optical fiber allows endomicroscopy to work at depth in living organisms and give live diagnostic information with minimal invasion.

Label-free nonlinear optical imaging techniques, providing a non-invasive approach for visualization of biomolecules, has proven to be a powerful tool for cancer research^1–6^. With the help of deep learning, these approaches can create virtual stained image, such as in coherent anti-Stokes Raman scattering (CARS), second harmonic generation (SHG), and two-photon excited auto-fluorescence (TPEF) modality^7–10^, bypassing the standard histochemical staining process^11–14^. To realize label-free imaging, the current microscopes either are coupled with standard-sized optical elements into a rigid needle endoscope with gradient index (GRIN) lenses^15–17^, or use fiber miniaturized resonant device to achieve fiber-scanning^18–20^. Both approaches increase equipment complexity and consequently manufacturing cost, and significantly enlarge the endoscope diameter and increase risk of tissue damage during diagnosis, limiting their clinical applications.

An alternative solution is utilizing coherent fiber bundle (CFB). CFB typically consists of thousands of cores, arranged in a honeycomb structure, with a common cladding. Each core acts as a pixel that individually transmits intensity imaged in near-field from the distal fiber end to the observer at the proximal fiber end. Various optical techniques have been successfully integrated with CFB, such as light-field imaging^21^, and holography^22–24^, micro manipulation^25,26^ and two-photon imaging^27,28^. The main challenge of application of CFB in clinics is that the honeycomb structure of CFB results in artifacts and limits the spatial resolution, which interfere with the identification of pathological tissue and hinders diagnosis. Therefore, elimination of honeycomb artifacts and improving resolution of CFB image is an urgent demand for label-free imaging using fiber-based endoscopy.

Conventional depixelation methods like Fourier domain filtering^29^ and interpolation^30^ can remove the honeycomb artifacts, but cannot improve resolution. Optimization methods, such as maximum a posteriori estimation^31^ and compressive sensing^32,33^, could improve the imaging quality by introducing prior information, but involve a time-consuming iterative procedure. With the multi-frame method, a sequence of images is captured with displacement or rotation of the fiber to add information^34–36^, however, the extra image registration increase complexity to the imaging system. Recently, deep learning has been shown to offer nonlinear fitting abilities in image regression problems^37–42^. Thus, learning-based methods were applied to CFB imaging for depixelation and resolution enhancement^43–46^. These works have limited sample types and numbers, however, which constrains the generalization capability to medical diagnosis.

In this paper two questions are to be solved: (1) the possibility of real-time reconstruction and resolution enhancement for fiber endoscopic images, and (2) whether the enhanced resolution helps the discrimination of tumor from healthy tissue.

For the first question, a display-CFB-sensor imaging system is set up to collect labeled CFB images. Then, they are put into a customized reconstruction network, which consists of a U-Net and an enhanced deep super-resolution (EDSR), first. The resolution enhancement network enables removing the honeycomb artifacts and EDSR reconstructs high-resolution features beyond the physical limitations of the CFB. In the second part, enhanced CFB images are fed into a classification neural network based on Visual Geometry Group-19 (VGG-19), which gives a prediction of tissue, whether it is a tumor or not. The binary classification is implemented on 9 kinds of brain tumor, most of which achieve excellent results. For glioblastoma, one kind of highly aggressive brain tumor, the reconstructed high-frequency features help increase its classification accuracy (prediction correct rate of all tissues) from 90.8 to 95.6%.

Based on the two points above, we proposed an end-to-end tumor diagnosis scheme using artificial intelligence (AI) technology to provide both high-resolution endoscopic imaging and tumor prediction results (Fig. 1b). Our approach adopts single-shot manner so that no scanning parts and post-processing algorithms are required, which is an advantage to realize real-time diagnosis. Another unique selling point is that, due to the simple and compact structure, the low cost of our endoscope makes a single-use-probe in clinics very promising, and the risk of post-surgical cross infection is minimized, therefore. The novel fiber-based diagnosis scheme dispenses the cumbersome process of biopsy and mitigates discomfort caused by invasion, and is thus low-cost and friendly to both of patients and physicians. A paradigm shift from the conventional diagnosis based on histochemical staining to a real-time and in situ diagnosis using label-free endomicroscopic imaging is achieved.

## Results

### Optimal working distance for lensless endoscope

**Figure 2:**
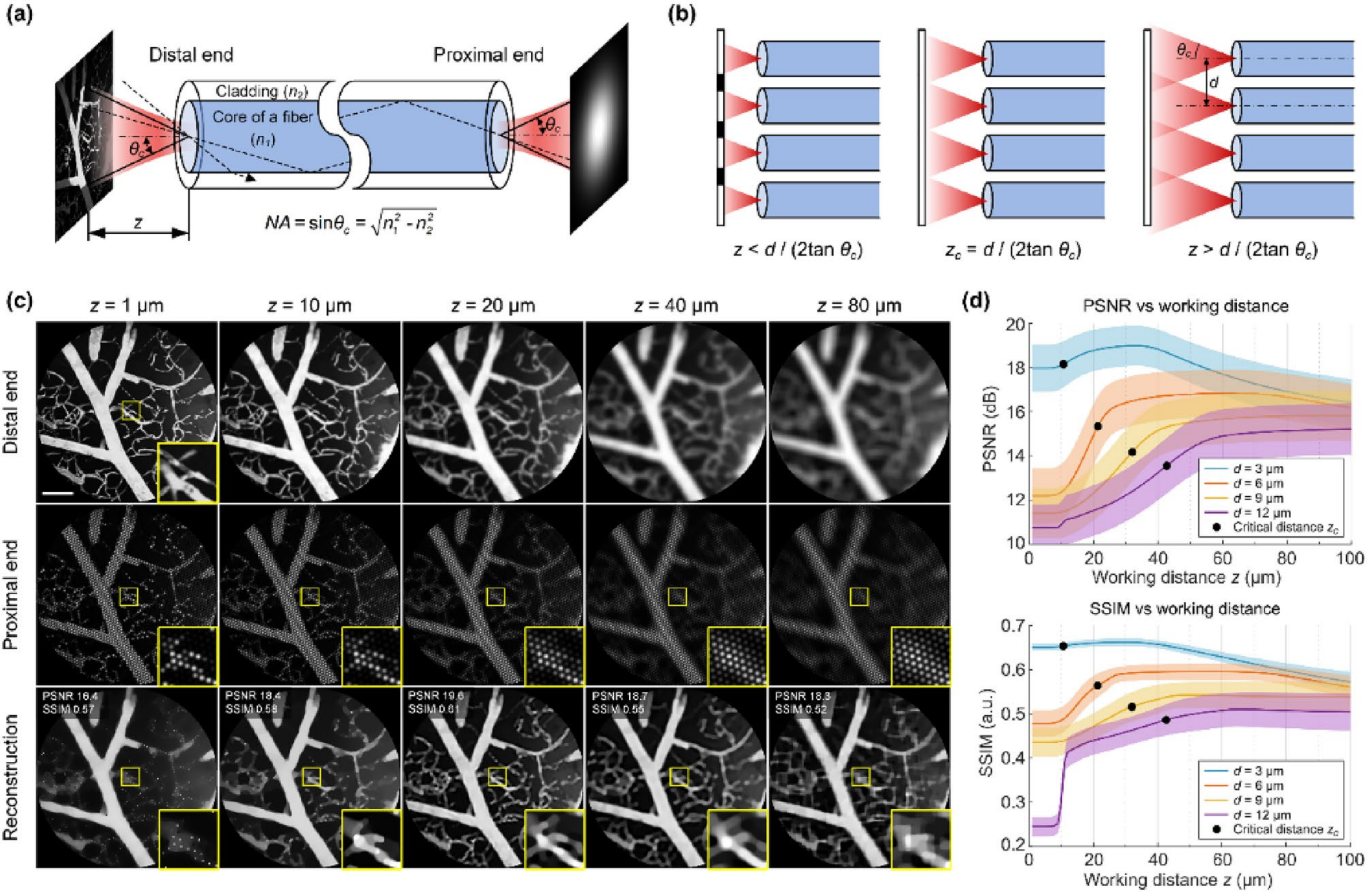
Optimal working distance for reconstruction. (**a**) Schematic of the transmission of an image by optical fiber as a single pixel. The working distance z and NA of fiber determine a cone space in which light can be coupled into fiber. (**b**) The situation that CFB gather light from sample under different working distance z. Areas that can be illuminated by and that can contribute to the intensity in each fiber core for different working distances. (**c**) The images at two ends of CFB and reconstructed images under different working distances. The reconstruction method is compressive sensing with TV regularization. Scale bar: 30 μm. Free space image propagation and deterioration by the CFB are realized numerically. (**d**) PSNR and SSIM distributions at varying working distances over 10 images of mouse cortical vasculature. Plotted are the mean (line) and 95% confidence interval (shading). The black dots indicate the critical distances.

Lensless endoscopes collect light directly from the distal CFB facet, enabling tiny probes to minimize invasion. As a near-field imaging manner, it requires no modulation and scanning, which are common methods of far-field imaging^22,23,47^. Working distance is an important parameter for CFB imaging in lensless mode. The optimal working distance is related to the core spacing and numerical aperture (NA) of the fiber. The NA of a fiber is defined as the sine of the largest acceptable angle *θ*_*c*_ that an incident ray can have for total internal reflectance in the fiber core (Fig. 2a). The NA can be calculated according to the refractive indices of core and cladding. It determines a cone space in which light can be coupled into fiber. For a CFB with *NA* = sin *θ*_*c*_, and core spacing *d*, when a sample is very close to the fiber facet, that is *z* < *d */ (2tan *θ*_*c*_), the regions which can be coupled into fiber cores does not completely cover the sample, which causes loss of information (Fig. 2b). When *z* = *d* / (2tan *θ*_*c*_), the light from the sample just happens to be coupled into the fiber. This working distance is critical distance *z*_*c*_ without information loss. When a sample is further away from the fiber facet, that is *z* ≫ *d* / (2tan *θ*_*c*_), a blurred observation results (Fig. 2b). Therefore, an optimal working distance is necessary to high-resolution imaging.

Here we use the parameters of a commercial CFB (Sumita HDIG) to analyze the optimal working distance, where the core spacing *d* = 3.0 μm and the acceptable angle *θ*_*c*_ = 8°. The critical distance is *z*_*c*_ = 10.7 μm. A multiphoton microscopic image of mouse cortical vasculature^48^ is chosen as true scene. We show the two ends of CFB and reconstructed images at five different working distances: 1 μm, 10 μm, 20 μm, 40 μm and 80 μm (Fig. 2c). Reconstruction is performed using compressive sensing (CS) with total variation (TV) regularization. When the working distance is 1 μm, the true values of the region between the cores, called dead-space, cannot be recovered. As the working distance increases, the information that was originally in dead-space can be collected by the adjacent cores, so that the recovery become possible. However, as the distance increases, the image at distal facet is blurred; this in turn makes it more difficult to recovery details. To disclose the relationship between optimal working distance and core spacing, we calculate the reconstructed image quality under different working distance and different core spacing to find the optimal working distance. Peak signal to noise ratio (PSNR) and structural similarity index measure (SSIM) are adopted for quantitative evaluation of image quality. The results show reconstruction can achieve optimum quality when the working distance is about 3*z*_*c*_ = 1.5*d* / tan *θ*_*c*_ (Fig. 2d). To attain the optimal working distance in practice, a layer of glass or polymer can be attached to the tip of the fiber. Then the object contacts with the cover layer so that the working distance can be fixed at the optimal distance.

### Resolution enhancement model based on U-Net + EDSR

**Figure 3:**
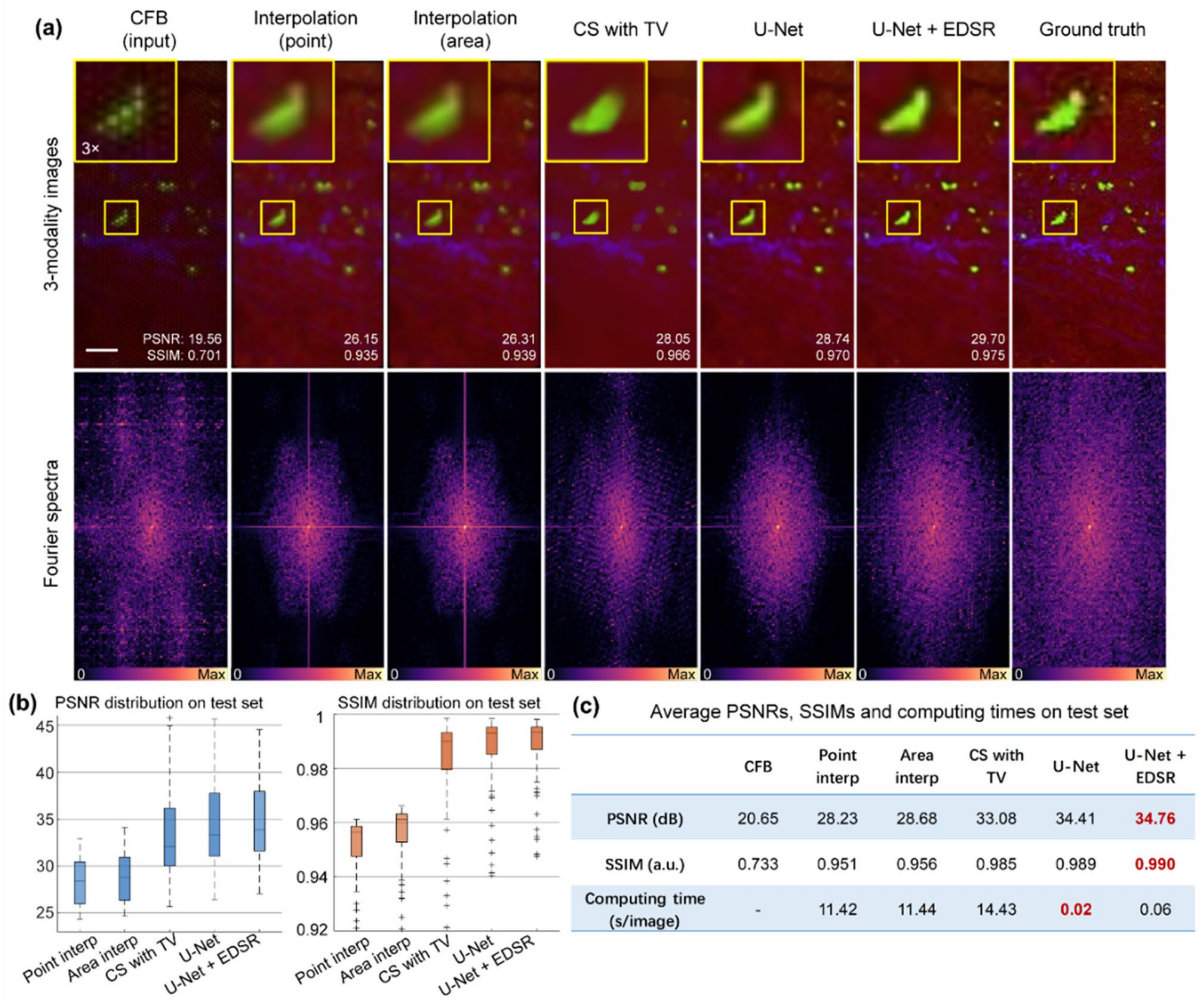
Image enhancement for the synthetic CFB images. (**a**) Comparison of enhanced images with ground truth by different methods in spatial and frequency domain. Scale bar: 20 μm. (**b**) Quantitative evaluation of the image quality of different methods in terms of PSNR and SSIM. Box plot: center line, median; box limits, upper and lower quartiles; whiskers, 1.5 × interquartile range; plus sign, outliers. (**c**) Average PSNRs, SSIMs and computing times of different methods. The U-Net + EDSR provides best image quality and fast computation.

A simple way to generate datasets is to synthesize CFB images from GT images. Here we adopt label-free multiphoton images as GT to synthesize the CFB images. The images were obtained using a multi-modal microscope, image modalities are CARS, TPEF, and SHG. The three modalities are combined into single RGB image. The lateral resolution is 1 μm. Image size is 208 × 104 pixels. A CFB imaging model is applied to generate pixelated images^49^. The optic fiber model of Sumita HDIG is simulated for dataset generation, where the core diameter is 2.0 μm and core spacing is 3.0 μm. The well-trained network was applied to the test set, which contains 200 RGB images of 9 tumor and 2 non-tumor types. We compare the results for the methods of point interpolation, area interpolation, CS with TV regularization. The instance shows that the learning-based methods are superior to all others (Fig. 3a). Moreover, the U-Net + EDSR configuration shows edges more clearly than U-Net-only configuration. The U-Net architecture could learn the features at different resolution scales, but U-Net lacks deeper layers to learn complex and variable features in each scale. EDSR consists of deep residual blocks, so connecting EDSR with U-Net can make up for the limited ability of network characterization at high resolution scale. The enhanced image by U-Net + EDSR has prominent target features, which can help a doctor discriminate tissue type intraoperatively. By quantitatively analyzing the quality of reconstruction on 200 images in test set, U-Net + EDSR has the highest average PSNR and SSIM, and has more centralized distributions than U-Net (Fig. 3b). The computing time is much faster than interpolation and CS methods (Fig. 3c).

### Resolution enhancement for experimentally acquired images

**Figure 4:**
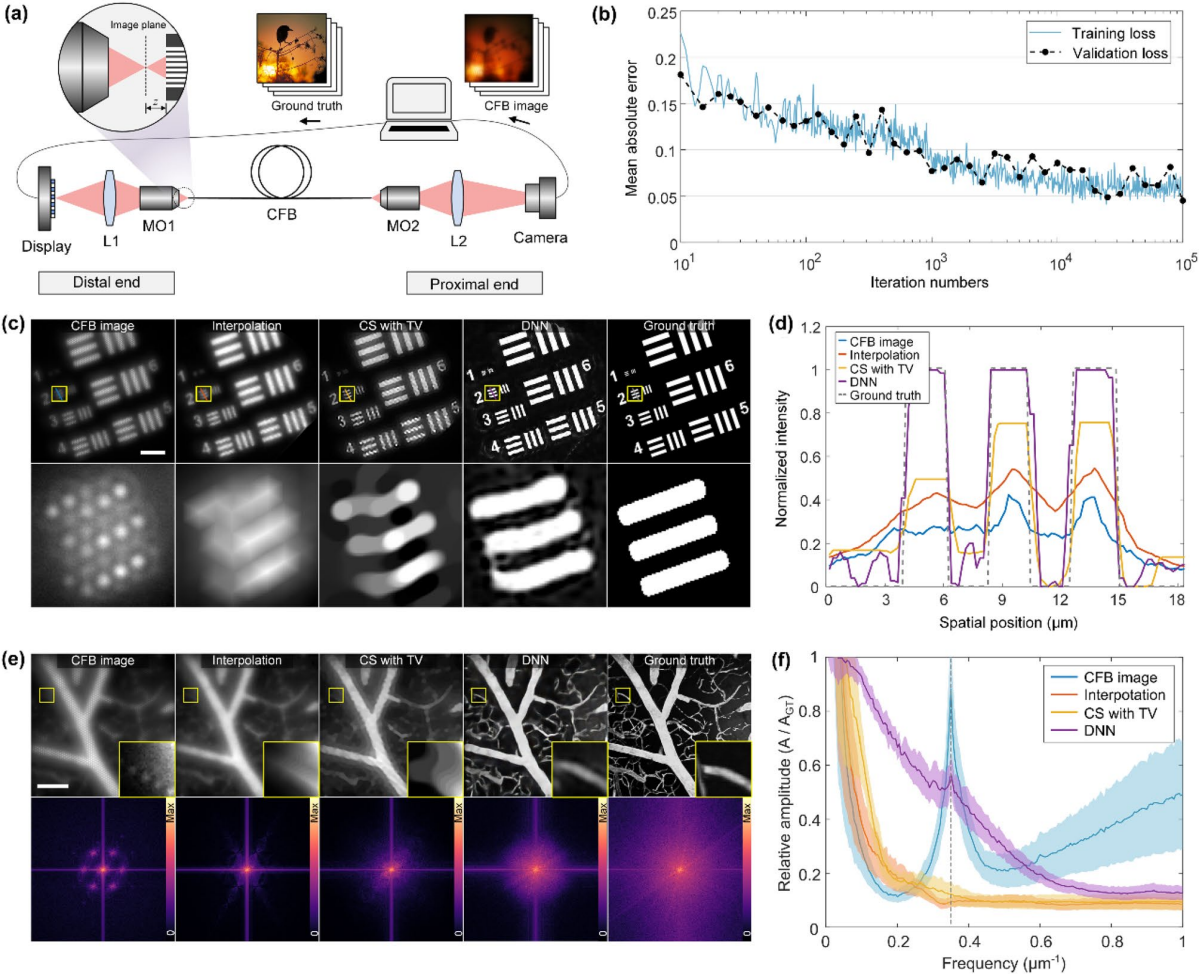
Image enhancement for the real CFB. (**a**) Experimental setup for dataset acquisition. The displayed images are projected to the optimal working distance z = 20 μm in front of the fiber fac images et. L, lens; MO, microscopic objective. (**b**) The change of loss function during training process. (**c**) Comparison of imaging results between different methods for resolution chart. Scale bar, 50 μm. (**d**) Cross section on Group-2 for each method. (**e**) Comparison of frequency domain characteristics of different image enhancement methods. The closeups show the details of tiny vessels. Scale bar, 40 μm. The corresponding spectrum maps are shown in the bottom of spatial images. (**f**) Radial normalized amplitude of spectrums. Plotted are the mean (line) and 95% confidence interval (shading) over 10 images. The dashed line indicates the core sampling frequency.

Defects in real CFBs make the actual images deviate from simulations. For example, irregular core shape and nonuniform refractive index may cause the inner-core coupling or excite cladding modes. These factors can lead to failure of network prediction for real CFB images. To address the problem, a display-CFB-sensor imaging system is setup to obtain pairs of real CFB images and GT images directly (Fig. 4a). DLP LightCrafter Display 4710 is adopted as the display and the Thorlabs Quantalux is adopted as the image sensor. A neutral density filter is used to reduce the light intensity to that comparable to the fluorescence. However, the light intensity is moderately increased to improve the image signal-to-noise ratio (SNR) for training set collection. Then the network can better learn the mapping between the fiber image and the original image without noise interference. The screen is projected onto the CFB (Sumita HDIG) facet using a 40 × objective and tube lens. The GT images are projected 30 μm away from the facet in accordance with the results in Fig. 2d. The distal CFB facet is then imaged onto a camera with 2.2 μm pixel pitch. The magnification is adjusted to × 2.7 so that the CFB occupies the same pixels number with GT image. We train the network for 10^5^ iterations, and the validation loss always remains the same level with training loss (Fig. 4b), which shows the network has good generalization performance.

A customized resolution chart is displayed on the screen to experimentally test the imaging resolution and contrast. The group number indicates the line width in pixels. According to the pixel pitch of display and objective magnification, the minimum line width in the projected image is 1.15 μm. For all the reconstruction methods, Group-2 can be resolved (Fig. 4c), thus an upper bound for the resolution is 2.3 μm or 217 lp/mm. This is better than the core-to-core distance of 3 μm and achieved by the increased working distance. While the increased working distance decreases the contrast initially, the EDSR enables contrast enhancement again. The cross sections of Group-2 show the imaging contrast (Fig. 4d). Defining the contrast as (*I*_*max − *_*I*_*min*_) / (*I*_*max*_ + *I*_*min*_) × 100%, where the *I*_*max*_ and *I*_*min*_ represent the average intensity of the white and black regions respectively. Then the contrast of the CFB image, interpolation, CS and DNN methods are 65.6%, 72.1%, 75.2%, and 86.3% respectively.

Furthermore, the frequency domain characteristics of reconstructed images are analyzed. The validation on mouse vasculature image shows that the DNN method can recover the most high-frequency components (Fig. 4e). To explicitly compare the frequency component that can be recovered by various methods, the amplitude spectra are averaged along the radial coordinate first and normalized to the spectral amplitude of the GT, for ten different images. The results are plotted in frequency-amplitude-curves (Fig. 4f). Note that the curve peak of the CFB image indicates the sampling frequency of the fiber core pitch at 0.33 μm^−1^, which is illustrated by the dashed line. According to Nyquist-Shannon sampling theorem, spectrum aliasing occurs when the signal frequencies exceed half of the sampling frequency. Interpolation methods only flatten the frequency curve but do not introduce high frequency components. The CS method introduces prior information through TV regularization, and high frequency components are slightly improved. However, TV regularization with a scalar weight is based on spatially invariant assumptions, which make it difficult to handle both homogeneous features and regions with rich details. In contrast, the DNN method can learn various features from the dataset, so that it can adaptively restore variable image features.

### Influence of image resolution on tumor classification results

Fluorescence imaging for tissue provides rich information for tumor diagnosis and indicate tumor margin^50^, degree of tumor progression^51^ and other pathological features for fine-grained analysis^52^. However, all these diagnostic techniques rely on high-resolution fluorescence images, which are often difficult to obtain in medical practice.

**Figure 5:**
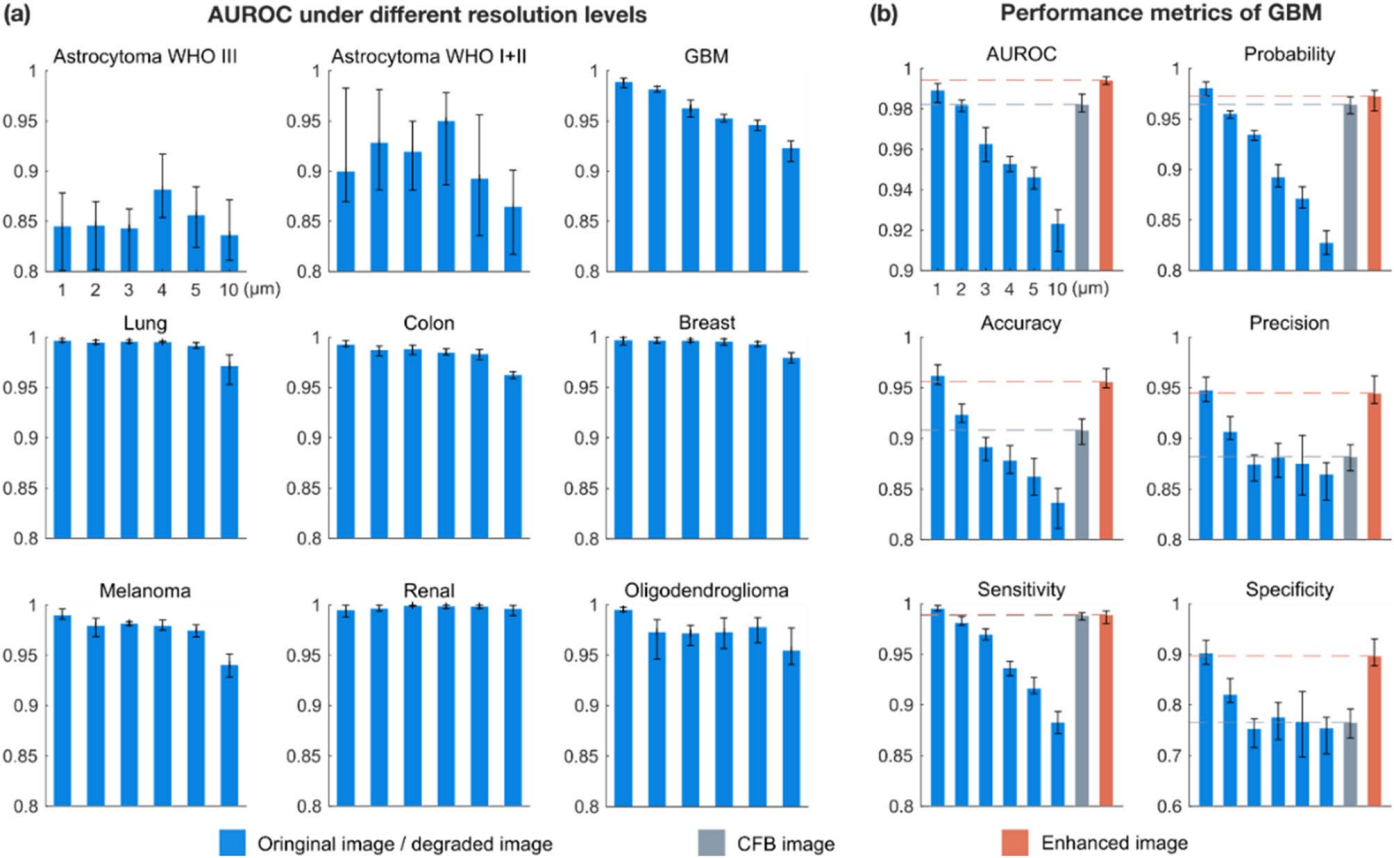
Comparison of classification performance for different image types. (**a**) AUROC values of the classification networks trained on different resolution levels. (**b**) The classification performance for GBM. The classification networks are individually trained and predicted for each data type CFB images and resolution enhanced images. All the results are based on TPEF modality.

In this work the dependence of tumor recognition on image resolution will investigated. For the tumor delineation, only binary discrimination of tumor and non-tumor is necessary. If the classification results are not sensitive to resolution, a low resolution and large field of view endoscope can be adopted for rapid tumor screening. Otherwise, high resolution imaging technology is required. We applied Gaussian filters with different kernel sizes to reduce the resolution for the TPEF images of biopsies of human brain towards malignant and benign tumors. The full width at half maximum of Gaussian filter is used to represent the resolution of degraded images. A VGG-19^53^ classification network is trained on the resolution of 1 μm (original resolution), 2 μm, 3 μm, 4 μm, 5 μm and 10 μm (Fig. 5a). The area under receiver operator characteristic curve (AUROC)^54^ is used for each test dataset as the performance metric. For each case, the training process is repeated for 5 times with different patients randomly chosen for training, in order to reduce the error caused by the randomness during data preparation and training. The upper and lower limits of error bar indicate the maximum and minimum achieved values.

For most tumors except glioblastoma (GBM), the AUROCs show low correlation with resolution. The problem of overfitting appeared in the training process of astrocytoma WHO I + II and III (Supplementary Fig. 1). For anaplastic oligodendroglioma WHO III, only at the 1 μm resolution, AUROC is stable and has a high level. This tumor type may have more high-frequency features (Supplementary Fig. 2) so that the diagnosis strongly dependents on high resolution images.

For GBM, the AUROCs show a nearly linear decrease with resolution. Since GBM is one of the most aggressive and lethal brain tumors, this highlights the necessity for high resolution imaging systems. Furthermore, GBM can be used to verify if the U-Net + EDSR network was truly able to retrieve high spatial frequency information by comparing classification results for original CFB images, enhanced CFB images and microscope images with different resolutions.

### Efficient improving GBM classification performance using resolution enhancement network

GBM is a highly aggressive type of brain tumor, so early diagnosis and treatment are of great significance for prolonging the life span of patients. Here we use six metrics of AUROC, probability, accuracy, precision, sensitivity, and specificity to evaluate the effect of using resolution enhancement network on classification performance. We individually trained networks on microscopic images, CFB images and resolution enhanced images, and the classification results on different resolution are shown for comparison (Fig. 5b). In all metrics, the resolution enhanced images have better performance compared to the CFB images. This verifies the proposed resolution enhancement network can efficiently improving GBM classification performance. The average accuracy of microscopic images, CFB image and enhanced image are 96.2%, 90.8%, 95.6%, respectively. In this case, the honeycomb artifacts of CFB images deteriorate some characteristics of tumor morphochemistry and reduce the classification performance slightly. Then the resolution enhancement process can reconstruct features and improve classification accuracy to the same level as the microscopic image.

## Discussion

Fast, precise, and minimally invasive tissue classification are crucial in cancer treatment. The current approach based on biopsies and histopathological analyses has some major drawbacks. It requires an additional intervention, which can cause trauma such as internal bleeding. It consists of a labor-intensive process chain lasting several hours to days. In addition, it exists the possibility to have recovered ill-suited tissue resulting in an inconclusive histopathological analysis. Recently fast and all optical, virtual staining and tissue classification has been demonstrated using DNNs. Translation into clinical practice requires minimally invasive approaches.

Here we discussed the usability of commercial coherent fiber bundles for direct imaging in conjunction with DNN-based super-resolution to enable a simple, robust, and cheap endoscopic system enabling single-use-probes. The method is based on applying the CFB in a defocused manner, to capture information from dead spaces and allow for compressive reconstruction. The enhancement in resolution beyond the fiber core spacing is demonstrated in simulation as well as on real images through a CFB, resulting in higher contrast and generally more recovered features high spatial frequency. We ensured generalization by training on random images from the ImageNet database unrelated to the later application.

Furthermore, we have shown that two-photon exited fluorescence microscopy in conjunction with a standard VGG-19 network is well suited for binary tissue classification over a variety of different tumor types and that classification results for glioblastoma greatly depends on optical resolution. Thus, we used GBM as a proxy to investigate if clinically relevant data can be recovered via the U-Net & EDSR network. We found an increased performance over several different metrics which was similar to ex vivo microscope images with 1 μm resolution. This highlights the potential of this approach towards in vivo diagnostics.

In conclusion, an end-to-end tumor diagnosis scheme is proposed by combining the reconstruction network and the classification network, to provide both high-resolution endoscopic imaging and tumor recognition. Our approach adopts single-shot manner so that no scanning parts and post-processing algorithms are required, which is an advantage to realize real-time imaging and makes intraoperative diagnosis possible. Another unique selling point is that, due to the simple and compact structure, the low cost of our endoscope makes a single-use-probe in clinics promising, and the risk of post-surgical cross infection is minimized, therefore. The novel fiber-based diagnosis scheme dispenses the cumbersome process of biopsy and mitigates discomfort caused by invasion, and is thus friendly to both of patients and physicians. A paradigm shift from the conventional diagnosis based on histochemical staining to a real-time and in situ diagnosis using label-free endomicroscopic imaging is achieved.

Additionally, it should be noted that the approach for enhancing CFB images is not limited towards brain cancer discrimination, to non-linear microscopy nor to lens-less CFB schemes. For example, the approach can easily be translated to holographic imaging. By selecting optimal object-image distance in both distal end and optimal end, a CFB can be used for cell size measurement^55,56^. The approach can also combine with optical coherence tomography (OCT), virtual staining and other medical imaging techniques with high requirements for abundant feature information. In order to advance the translation to the clinic, further experimental analysis on bulk tissue will be investigated next as well as the use on linear auto-fluorescence for an even simpler setup. Lastly, since the explainable AI has made some progress in real-world applications^57,58^, the future research will focus on the mechanisms of tumor classification by DNN, and improve the network to provide more robust, reliable and abundant information, such as the degree of the lesion and lesion area, so that deep learning methods can provide trusty means of medical diagnosis.

## Methods

### CFB-based imaging model

The CFB translates the spatial intensity distribution on the distal facet to the proximal facet in a degraded, pixelated manner. Considering a sample placed at a distance *z* from the distal facet, each fiber core couples the light within its acceptance angle, implementing a weighted sum of the original image. The image degradation can be modeled as convolution with a point spread function (PSF), consisting of three parts: distance attenuation term, source divergence angle and facet coupling efficiency. The distance attenuation term follows inverse-square law, and considering the fiber critical angle, rays with an incident angle greater than the critical angle *θ*_*c*_ cannot be coupled into the fiber. The distance attenuation term could be formalized as:

1 $$I_{dist} \left( {\vec{r};z} \right) = \left\{ \begin{gathered} \frac{{z^{2} }}{{z^{2} + \left| {\vec{r}} \right|^{2} }}, \, \theta \le \theta_{c} \hfill \\ 0, \, \theta > \theta_{c} \, \hfill \\ \end{gathered} \right.,$$

where $\theta = \arctan \left( {{{\left| {\vec{r}} \right|} \mathord{\left/ {\vphantom {{\left| {\vec{r}} \right|} z}} \right. \kern-\nulldelimiterspace} z}} \right)$. The facet coupling efficiency depends on the collection aperture of the fiber. An approximation model for the facet coupling efficiency is a Gaussian distribution, which can be parameterized as follow:

2 $$I_{\sigma } \left( {\vec{r};\sigma } \right) = \exp \left[ {{{ - \left| {\vec{r}} \right|^{2} } \mathord{\left/ {\vphantom {{ - \left| {\vec{r}} \right|^{2} } {2\sigma^{2} }}} \right. \kern-\nulldelimiterspace} {2\sigma^{2} }}} \right],$$

where *σ* denotes the width of the Gaussian function. When the full width at half maximum (FWHM) of the Gaussian distribution is equal to the fiber core diameter *d*, *σ* has the value ${d \mathord{\left/ {\vphantom {d {2\sqrt {2\ln \left( 2 \right)} }}} \right. \kern-\nulldelimiterspace} {2\sqrt {2\ln \left( 2 \right)} }}$. Assuming the light source has uniform distribution in all angle, the total PSF could be modelled as:

3 $${\text{PSF}}\left( {\vec{r};z,\sigma } \right) = I_{\sigma } \left( {\vec{r};\sigma } \right) * I_{dist} \left( {\vec{r};z} \right),$$

where “*” denote convolution sign. Then each fiber cores samples the intensity at distal facet:

4 $$Y_{i} = \left[ {{\text{PSF}}\left( {\vec{r};z,\sigma } \right) * X\left( {\vec{r}} \right)} \right] \cdot \delta \left( {\vec{r} - r_{i} } \right),$$

where *r*_*i*_ is the position vector of the core centers. *Y*_*i*_ represents the *i*th downsampling measurement for the high-resolution image *X*. Then the fiber bundle conveys the sampled intensities to the proximal end. For single mode cores, only the LP_01_ mode can be transmitted over optical fibers so that all the cores have the same relative intensity distribution at the proximal facet. The LP_01_ mode of optical fiber is often expressed approximately by the Gaussian field^59^. Then applying the convolution again to form the observed honeycomb-like image:

5 $$I_{honeycomb} \left( {\vec{r}} \right) = \frac{1}{{2\pi \omega^{2} }}\exp \left[ { - {{\left| {\vec{r}} \right|^{2} } \mathord{\left/ {\vphantom {{\left| {\vec{r}} \right|^{2} } {2\omega^{2} }}} \right. \kern-\nulldelimiterspace} {2\omega^{2} }}} \right] * \sum\limits_{i}^{N} {Y_{i} \left( {\vec{r}} \right),}$$

where deviation *ω* is the equivalent mode field radius, which is related to the fiber parameters.

### Compressive reconstruction

The true resolution of the honeycomb-like image is the number of fiber cores, which is usually much less than the number of sensor pixels. To reconstruct a high-resolution image from the CFB image is an ill-posed problem. Compressive sensing (CS) is a typical method to deal with such problems. Compressive sensing is a powerful signal reconstruction framework and provides complete theoretical support for image reconstruction. It states a given signal can be reconstructed accurately with fewer samples or measurements, which is not necessary to satisfy the Nyquist's sampling theorem^60^. CS theory indicates the conditions for accurate reconstruction are sparsity and incoherence^61^. Sparsity means there are many zero-valued elements in the signal itself or in some transform domain. Incoherence means that sensing matrix and representation matrix are uncorrelated. Natural images are sufficiently sparse with its representation in the gradient domain^62^ or wavelet domain^63^. In CFB imaging, the general image degradation can be expressed as convolution form. CFB imaging can approximately satisfy the compressed sensing condition and achieve high-quality reconstruction.

Since the valid measurements for CFB imaging is the core intensities, the CFB image can be represented by integrating each intensity in the core and rearrange them into 1-dimensional (1D) vector $\vec{Y}$. If representing the original image as a 1D vector $\vec{X}$ in lexicographical order, then Eq. (4) can be rewritten in matrix–vector form:

6 $$\vec{Y} = DC\vec{X} + \vec{E}$$

where $\vec{E}$ is the additive noise, *C* is convolution operation, and *D* is the downsampling operation. All linear manipulations can be simplified into CS literature form to:

7 $$\vec{Y} = W\vec{X} + \vec{E}$$

where *W* operation combines the convolution and down-sampling operation into a single operation. Assuming the fiber bundle has *M* cores, and the camera has *N* pixels, where *M* < *N*, then $\vec{Y}$ is the *M* × 1 vectorized observation, $\vec{X}$ is the *N* × 1 vectorized object, and *W* is a *M* × *N* matrix. Obviously, this is an underdetermined system. The traditional solution for this problem is the least squares method, which is to solve the following optimization problem:

8 $$\hat{X} = \arg \mathop {\min }\limits_{{\vec{X}}} \left\| {W\vec{X} - \vec{Y}} \right\|_{2}^{2}$$

However, additive noise in measurements greatly affects the accuracy of the results. Thus, it is necessary to introduce a regularization term to stabilize the solution. Then the optimization problem becomes:

9 $$\hat{X} = \arg \mathop {\min }\limits_{{\vec{X}}} \left\{ {\left\| {W\vec{X} - \vec{Y}} \right\|_{2}^{2} + \tau \Phi \left( {\vec{X}} \right)} \right\}$$

where *τ* is a coefficient that balances the regularization term and the data fitting term and Φ is the regularization term representing the prior. Reconstructions can be performed using the two-step iterative shrinkage/threshold (TwIST) algorithm^64^ with total variation (TV) regularization.

### Data acquisition

Our analysis of resolution enhancement and tumor classification based on a multiphoton image set, which comes from Uckermann’s et al. paper^65^. It includes Coherent anti-Stokes Raman Scattering (CARS), Two-Photon Excited Fluorescence (TPEF), and Second Harmonic Generation (SHG) microscopy images on cryosections of brain tumors of 382 patients and 28 human non-tumor brain samples. The previous research verified the combined analysis of texture parameters of the CARS and TPEF signal is most suited for the discrimination of non-tumor brain versus brain tumors. The classification includes different tumor types (low- and high-grade astrocytoma, oligodendroglioma, glioblastoma, recurrent glioblastoma, brain metastases of lung, colon, renal, and breast cancer and of malignant melanoma), and demonstrate a correct rate of 96% (sensitivity: 96%, specificity: 100%) by using linear discriminant analysis (LDA) method ^66^.

We reproduce the classification using deep neural network (DNN) in the case of single modality: TPEF, two modalities: CARS & TPEF, and three modalities: CARS & TPEF & SHG. TPEF is chosen in single modality for comparison because of its high classification accuracy^65^ and straightforward implementation in a fiber probe^67^. In our case, the total number of patients used for training, validation and testing are 311, 33, and 37, respectively (see the patient distributions of each tumor type in Supplementary Table 1). For each type of tumor, we randomly assigned 2 patients with non-tumor to participate in classification training. We use accuracy, sensitivity (correct rate of tumor) and specificity (correct rate of non-tumor) to evaluate the overall classification performance. The results show the single modality has a correct rate of 98.2% (sensitivity: 97.3%, specificity: 100%), which has almost equivalent performance with multi-modalities (Supplementary Table 2). The results verify the feasibility of clinical exploitation using two-photon fluorescence endomicroscopy systems.

### Network architecture and training process

The proposed network cascades a U-Net^68^ and a EDSR^69^ in sequence (Supplementary Fig. 3). The U-Net part consist of a series of down sampling and up sampling blocks to learn the features at different resolution scales. We remove the batch normalization layers in both networks, since they get rid of range flexibility from networks by normalizing the features. For the EDSR part, the network is mainly composed of residual blocks in series. The additional scaling layer in the residual block of the EDSR helps to stabilize the training progress. A convolution layer is used to extract features at the beginning and the end of all the residual blocks, respectively. A skip connection connects these two convolution layers. Finally, the image is output through a convolution layer. All convolutional layers use filters of size 3 × 3. Since there is no need to increase the image size in our case, we remove the upsample layer from the original model. The depth (the number of residual blocks) is 32 and feature number is 256.

The loss function is evaluated on both pixel-wise and feature-wise metrics. The mean absolute error is calculated as pixel-wise metric. The pretrained VGG-16 is used to define the perceptual loss function that measures perceptual differences in output and ground truth (GT) label. The total loss function is the sum of these two terms with an adjustable weighting coefficient.

We trained two networks for synthetic images and real images respectively. For the synthetic images, 10,000 images from multiphoton biopsies images of human brain tumors are randomly selected and then cropped to the size of 192 × 96. 9,500 of them are used for the training, 300 for the validation and 200 for the testing. For real images, we adopt 5,500 natural images from ImageNet^70^ as GT to display on the screen, which are scaled to 512 × 512 pixels for display. The minibatch size is 4. The learning rate is initialized to 10^−4^ for all layers and decreases by a factor of 0.5 for 2 × 10^3^ iterations. The training was run on a workstation with 32 AMD Ryzen 9 3950X 16-Core Processors and a NVIDIA RTX A6000 GPU.

## Supplementary Information

Supplementary Information.

## Data Availability

The datasets generated and/or analyzed during the current study are available from the corresponding author upon reasonable request.

## References

[1] Freudiger CW (2008). Label-free biomedical imaging with high sensitivity by stimulated Raman scattering microscopy. Science.

[2] Azarin SM (2015). In vivo capture and label-free detection of early metastatic cells. Nat. Commun..

[3] Traynor D (2021). Raman spectral cytopathology for cancer diagnostic applications. Nat. Protoc..

[4] Mazumder N (2019). Label-free non-linear multimodal optical microscopy—basics, development, and applications. Front. Phys..

[5] Placzek F (2020). Morpho-molecular ex vivo detection and grading of non-muscle-invasive bladder cancer using forward imaging probe based multimodal optical coherence tomography and Raman spectroscopy. Analyst.

[6] Papageorgiou EP (2018). Real-time cancer detection with an integrated lensless fluorescence contact imager. Biomed. Opt. Express.

[7] Bocklitz TW (2016). Pseudo-HE images derived from CARS/TPEF/SHG multimodal imaging in combination with Raman-spectroscopy as a pathological screening tool. BMC Cancer.

[8] Petersen D (2017). Virtual staining of colon cancer tissue by label-free Raman micro-spectroscopy. Analyst.

[9] Capitaine E (2018). Fast epi-detected broadband multiplex CARS and SHG imaging of mouse skull cells. Biomed. Opt. Express.

[10] Baugh LM (2017). Non-destructive two-photon excited fluorescence imaging identifies early nodules in calcific aortic-valve disease. Nat. Biomed. Eng..

[11] Rivenson Y (2019). Virtual histological staining of unlabelled tissue-autofluorescence images via deep learning. Nat. Biomed. Eng..

[12] Zhang Y (2020). Digital synthesis of histological stains using micro-structured and multiplexed virtual staining of label-free tissue. Light Sci. Appl..

[13] Li J (2021). Biopsy-free in vivo virtual histology of skin using deep learning. Light Sci. Appl..

[14] You S (2019). Real-time intraoperative diagnosis by deep neural network driven multiphoton virtual histology. NPJ Precis. Oncol..

[15] Llewellyn ME, Barretto RP, Delp SL, Schnitzer MJ (2008). Minimally invasive high-speed imaging of sarcomere contractile dynamics in mice and humans. Nature.

[16] Liang W, Hall G, Messerschmidt B, Li M-J, Li X (2017). Nonlinear optical endomicroscopy for label-free functional histology in vivo. Light Sci. Appl..

[17] Liang W (2020). Throughput-speed product augmentation for scanning fiber-optic two-photon endomicroscopy. IEEE Trans. Med. Imaging.

[18] Kim JK (2012). Fabrication and operation of GRIN probes for in vivo fluorescence cellular imaging of internal organs in small animals. Nat. Protoc..

[19] Trägårdh J (2019). Label-free CARS microscopy through a multimode fiber endoscope. Opt. Express.

[20] Lombardini A (2018). High-resolution multimodal flexible coherent Raman endoscope. Light Sci. Appl..

[21] Orth A, Ploschner M, Wilson E, Maksymov I, Gibson B (2019). Optical fiber bundles: Ultra-slim light field imaging probes. Sci Adv.

[22] Kuschmierz R, Scharf E, Koukourakis N, Czarske JW (2018). Self-calibration of lensless holographic endoscope using programmable guide stars. Opt. Lett..

[23] Scharf E, Dremel J, Kuschmierz R, Czarske J (2020). Video-rate lensless endoscope with self-calibration using wavefront shaping. Opt. Lett..

[24] Leite IT, Turtaev S, Boonzajer Flaes DE, Čižmár T (2021). Observing distant objects with a multimode fiber-based holographic endoscope. APL Photonics.

[25] Sun J, Koukourakis N, Guck J, Czarske JW (2021). Rapid computational cell-rotation around arbitrary axes in 3D with multi-core fiber. Biomed. Opt. Express.

[26] Sun, J. et al. (2021) Lensless multicore-fiber microendoscope for real-time tailored light field generation with phase encoder neural network (CoreNet). *arXiv preprint *arXiv:2111.12758

[27] Andresen ER, Bouwmans G, Monneret S, Rigneault H (2013). Two-photon lensless endoscope. Opt. Express.

[28] Morales-Delgado EE, Psaltis D, Moser C (2015). Two-photon imaging through a multimode fiber. Opt. Express.

[29] Lee C-Y, Han J-H (2013). Integrated spatio-spectral method for efficiently suppressing honeycomb pattern artifact in imaging fiber bundle microscopy. Opt. Commun..

[30] Zheng, Z., Cai, B., Kou, J., Liu, W. & Wang, Z. in International Conference on Intelligent Autonomous Systems 771–779 (Springer, 2016).

[31] Shao J, Liao W-C, Liang R, Barnard K (2018). Resolution enhancement for fiber bundle imaging using maximum a posteriori estimation. Opt. Lett..

[32] Dumas J, Lodhi M, Bajwa W, Pierce M (2018). A compressed sensing approach for resolution improvement in fiber-bundle based endomicroscopy. Endoscopic Microsc. XIII.

[33] Dumas JP, Lodhi MA, Taki BA, Bajwa WU, Pierce MC (2019). Computational endoscopy—a framework for improving spatial resolution in fiber bundle imaging. Opt. Lett..

[34] Lee C-Y, Han J-H (2013). Elimination of honeycomb patterns in fiber bundle imaging by a superimposition method. Opt. Lett..

[35] Cheon GW, Cha J, Kang JU (2014). Random transverse motion-induced spatial compounding for fiber bundle imaging. Opt. Lett..

[36] Renteria C, Suárez J, Licudine A, Boppart SA (2020). Depixelation and enhancement of fiber bundle images by bundle rotation. Appl. Opt..

[37] Wang H (2019). Deep learning enables cross-modality super-resolution in fluorescence microscopy. Nat. Method.

[38] Rahmani B, Loterie D, Konstantinou G, Psaltis D, Moser C (2018). Multimode optical fiber transmission with a deep learning network. Light Sci. Appl..

[39] Rothe S, Zhang Q, Koukourakis N, Czarske J (2021). Intensity-only mode decomposition on multimode fibers using a densely connected convolutional network. J. Lightwave Technol..

[40] Kuschmierz R, Scharf E, Ortegón-González DF, Glosemeyer T, Czarske JW (2021). Ultra-thin 3D lensless fiber endoscopy using diffractive optical elements and deep neural networks. Light. Adv Manuf.

[41] Gataric M (2018). Reconstruction of optical vector-fields with applications in endoscopic imaging. IEEE Trans. Med. Imaging.

[42] Liu Y, Yuan H, Wang Z, Ji S (2020). Global pixel transformers for virtual staining of microscopy images. IEEE Trans. Med. Imaging.

[43] Shao J, Zhang J, Huang X, Liang R, Barnard K (2019). Fiber bundle image restoration using deep learning. Opt. Lett..

[44] Shao J, Zhang J, Liang R, Barnard K (2019). Fiber bundle imaging resolution enhancement using deep learning. Opt. Express.

[45] Ravì D, Szczotka AB, Shakir DI, Pereira SP, Vercauteren T (2018). Effective deep learning training for single-image super-resolution in endomicroscopy exploiting video-registration-based reconstruction. Int. J. Comput. Assist. Radiol. Surg..

[46] Ravì D, Szczotka AB, Pereira SP, Vercauteren T (2019). Adversarial training with cycle consistency for unsupervised super-resolution in endomicroscopy. Med. Image Anal..

[47] Yeminy T, Katz O (2021). Guidestar-free image-guided wavefront shaping. Sci. Adv..

[48] Teikari, P., Santos, M., Poon, C. & Hynynen, K. (2016) Deep learning convolutional networks for multiphoton microscopy vasculature segmentation. *arXiv preprint *arXiv:1606.02382

[49] Hughes, M., Vol. 2022 (MATLAB Community; 2020).

[50] Galli R (2019). Identification of distinctive features in human intracranial tumors by label-free nonlinear multimodal microscopy. J. Biophotonics.

[51] Vakoc BJ (2009). Three-dimensional microscopy of the tumor microenvironment in vivo using optical frequency domain imaging. Nat. Med..

[52] Lu MY (2021). Data-efficient and weakly supervised computational pathology on whole-slide images. Nat. Biomed. Eng..

[53] Simonyan, K. & Zisserman, A. (2014) Very deep convolutional networks for large-scale image recognition. *arXiv preprint *arXiv:1409.1556

[54] Zweig MH, Campbell G (1993). Receiver-operating characteristic (ROC) plots: A fundamental evaluation tool in clinical medicine. Clin. Chem..

[55] Li J, Dai L, Yu N, Wu Y (2021). Z-axis displacement measurement model of quasi-spherical cells based on microfluidics under lensless imaging. Meas. Sci. Technol..

[56] Li J, Dai L, Yu N, Li Z, Li S (2022). Lensless light intensity model for quasi-spherical cell size measurement. Biomed. Microdevice.

[57] Lim J, Psaltis D (2022). MaxwellNet: Physics-driven deep neural network training based on Maxwell’s equations. APL Photonics.

[58] Wetzstein G (2020). Inference in artificial intelligence with deep optics and photonics. Nature.

[59] Li, L. & Guo, F. in Information Optics and Photonics Technologies II, Vol. 6837 68370D (International Society for Optics and Photonics, 2008).

[60] Shannon CE (1948). A mathematical theory of communication. Bell Syst Technol. J.

[61] Donoho DL (2006). Compressed sensing. IEEE Trans. Inf. Theory.

[62] Krishnan, D. & Fergus, R. in Advances in neural information processing systems 1033–1041 (2009).

[63] Portilla J, Strela V, Wainwright MJ, Simoncelli EP (2003). Image denoising using scale mixtures of Gaussians in the wavelet domain. IEEE Trans. Image Process..

[64] Bioucas-Dias JM, Figueiredo MA (2007). A new TwIST: Two-step iterative shrinkage/thresholding algorithms for image restoration. IEEE Trans. Image Process..

[65] Uckermann O (2020). Label-free multiphoton imaging allows brain tumor recognition based on texture analysis—A study of 382 tumor patients. Neuro-oncol Adv.

[66] Fisher RA (1936). The use of multiple measurements in taxonomic problems. Ann. Eugen..

[67] Lukic A (2017). Endoscopic fiber probe for nonlinear spectroscopic imaging. Optica.

[68] Ronneberger O, Fischer P, Brox T (2015). 234–241.

[69] Lim, B., Son, S., Kim, H., Nah, S. & Mu Lee, K. (2017) in Proceedings of the IEEE Conference on computer vision and pattern recognition workshops 136–144

[70] Russakovsky O (2015). ImageNet large scale visual recognition challenge. Int. J. Comput. Vis..

