# Supplementary material for: Learned end-to-end high-resolution lensless fiber imaging towards real-time cancer diagnosis
Source: Sci Rep. 2022 Nov 7;12:18846. doi: 10.1038/s41598-022-23490-5 (PMC9640670; doi:10.1038/s41598-022-23490-5)
Supplement: Supplementary file 1 — Supplementary Information. [file 41598_2022_23490_MOESM1_ESM.docx]

**Learned end-to-end high-resolution lensless fiber imaging towards real-time cancer diagnosis**

**Supplementary information**

Jiachen Wu^1,2*^, Tijue Wang^1^, Ortrud Uckermann^3,4,9^, Roberta Galli^5^, Gabriele Schackert^3,9^, Liangcai Cao^2^, Jürgen Czarske^1,6,7,8,9*^, and Robert Kuschmierz^1,6,9*^

*^1^Laboratory of Measurement and Sensor System Technique, TU Dresden, Helmholtzstrasse 18, 01069 Dresden, Germany*

*^2^State Key Laboratory of Precision Measurement Technology and Instruments, Department of Precision Instruments, Tsinghua University, Beijing 100084, China*

*^3^Department of Neurosurgery, University Hospital Carl Gustav Carus, TU Dresden, Dresden, Germany*

*^4^Division of Medical Biology, Department of Psychiatry, Faculty of Medicine and University Hospital Carl Gustav Carus, TU Dresden, Germany*

*^5^Department of Medical Physics and Biomedical Engineering, Faculty of Medicine Carl Gustav Carus, TU Dresden, Germany*

*^6^Competence Center BIOLAS, TU Dresden, Dresden, Germany*

*^7^Excellence Cluster Physics of Life, TU Dresden, Dresden, Germany*

*^8^Faculty of Physics, School of Science, TU Dresden, Dresden, Germany*

*^9^Else Kröner Fresenius Center for Digital Health, TU Dresden, Germany*

**E-mail:* [*jiachen.wu@mailbox.tu-dresden.de*](mailto:jiachen.wu@mailbox.tu-dresden.de)*;* [*juergen.czarske@tu-dresden.de*](mailto:juergen.czarske@tu-dresden.de)*;* *[robert.kuschmierz@tu-dresden.de](mailto:robert.kuschmierz@tu-dresden.de)*

| Tissue type | Total images | Total patients | Training patients | Validation patients | Test patients |
| --- | --- | --- | --- | --- | --- |
| Astrocytoma WHO I + II | 739 | 13 | 9 | 2 | 2 |
| Astrocytoma WHO III | 5582 | 74 | 61 | 6 | 7 |
| Oligodendroglioma WHO III | 4373 | 41 | 33 | 4 | 4 |
| GBM | 9864 | 108 | 90 | 9 | 9 |
| Metastasis of colon cancer | 3405 | 25 | 20 | 2 | 3 |
| Metastasis of lung cancer | 6142 | 47 | 38 | 4 | 5 |
| Metastasis of renal cancer | 2825 | 20 | 16 | 2 | 2 |
| Metastasis of breast cancer | 3109 | 24 | 20 | 2 | 2 |
| Metastasis of melanoma | 3791 | 29 | 24 | 2 | 3 |
| Total (tumor tissue) | 39830 | 381 | 311 | 33 | 37 |
| Nontumor brain | 5344 | 18 | 14 | 2 | 2 |
| Total | 45174 | 399 | 325 | 35 | 39 |

**Supplementary Table 1. Composition of test and training set.** The number of patients is given for each tissue type.

**Supplementary Fig. 1.** The classification accuracy of Astrocytoma WHO III varies with iteration during training process. Overfitting occurred after 600 iterations.


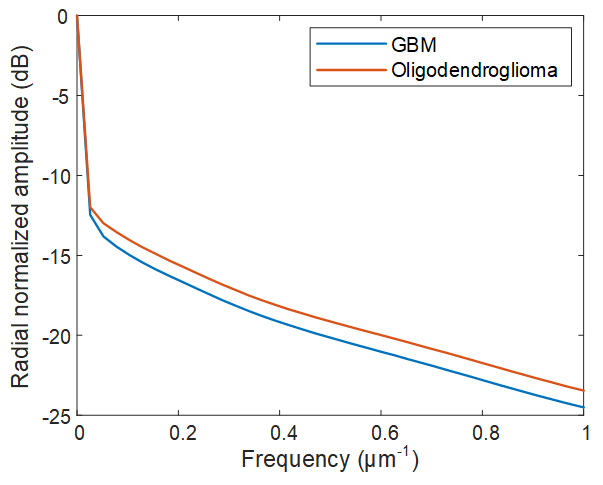


**Supplementary Fig. 2.** Comparison of the average frequency distribution of TPEF images between GBM and oligodendroglioma.


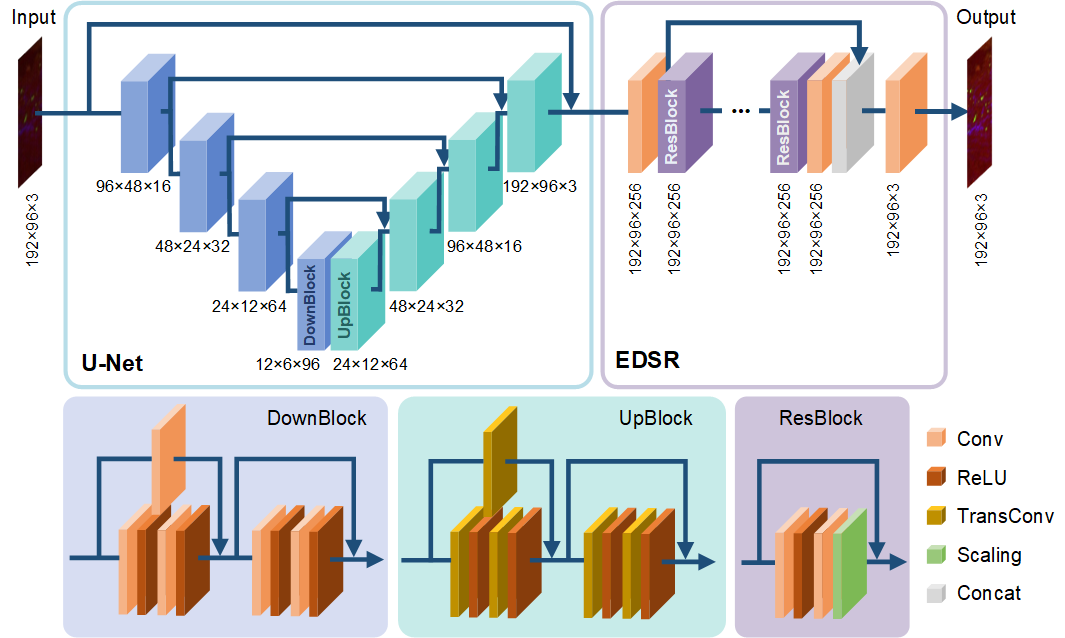


**Supplementary Fig. 3. The architecture of the proposed CFB image enhancement network.** It consists of two classical architectures: U-Net and EDSR. The difference from the original is the removed batch normalization layers in U-Net and removed up-sampling layer in EDSR.

| Methods (modalities) | Accuracy | Sensitivity | Specificity |
| --- | --- | --- | --- |
| LDA (TPEF) | 93% | 93% | 86% |
| LDA (CARS +TPEF) | 96% | 96% | 100% |
| DNN (TPEF) | 98.2% | 97.3% | 100% |
| DNN (CARS + TPEF) | 98.2% | 97.3% | 100% |
| DNN (CARS + TPEF + SHG) | 96.4% | 94.6% | 100% |

**Supplementary Table 2. Comparison of LDA and DNN methods on different modalities.** Accuracy (correct rate of all the tissue), sensitivity (correct rate of tumor), and specificity (correct rate of nontumor brain) are adopted for evaluating the performance of classification methods.
